# Cryoablation Utilizing the KODEX-EPD Mapping System Versus Conventional Cryoballoon Ablation in the Management of Patients With Atrial Fibrillation: A Literature Review and Meta-Analysis

**DOI:** 10.7759/cureus.59407

**Published:** 2024-04-30

**Authors:** Christopher R Meretsky, Vaishvik K Patel, Arshia Mahmoodi, Anthony T Schiuma

**Affiliations:** 1 Surgery, St. George's University School of Medicine, Great River, USA; 2 Medicine, St. George’s University, West Indies, GRD; 3 Orthopedic Surgery, Holy Cross Hospital, Fort Lauderdale, USA

**Keywords:** cardiac arrythmia, cryoballoon, kodex system, cryoablation, atrial fibrillation (af)

## Abstract

Atrial fibrillation (AF) is the most commonly encountered cardiac arrhythmia globally. AF is associated with different consequences, such as peripheral vascular embolism, stroke, dementia, heart failure, and death. Catheter ablation (CA) has become a reliable therapeutic option for symptomatic AF. Utilizing mapping systems in conducting cryoablation is supposed to improve pulmonary vein isolation (PVI) durability and overall treatment success rate. We performed a review of relevant articles. We formulated a search strategy as follows: (atrial fibrillation AND ("cryoballoon ablation" OR cryoablation) AND (KODEX-EPD AND KODEX OR mapping). Data were collected from Web of Science, PubMed, Cochrane Library, and SCOPUS databases. We assessed the efficacy, procedural characteristics, and safety of cryoablation using the KODEX-EPD mapping system versus conventional cryoablation. We demonstrated the superiority of cryoablation guided by the KODEX-EPD system as it was associated with a significantly lower recurrence rate after the procedure (RR = 0.61, P = 0.03). Furthermore, it allowed a significant reduction in the volume of contrast medium used during the procedure (MD = -20.46, P = 0.04) when compared to the conventional cryoablation. We found no significant difference between both procedures in terms of successful cryoballoon-based PVI (P = 1.00), procedural duration (P = 0.95), procedural complications (P = 0.607), fluoroscopic time (P = 0.36), and fluoroscopic dose (P = 0.16). The use of the novel KODEX-EPD mapping system in the cryoablation procedure was associated with a significant reduction of the volume of contrast medium use and the recurrence rate compared with the conventional cryoablation while preserving similar efficacy, safety profile, and procedure time.

## Introduction and background

Atrial fibrillation (AF) is the most commonly encountered cardiac arrhythmia affecting about 33 million patients globally [1]. The number of people suffering from AF is predicted to increase by two or three times by 2050 [2,3]. It usually occurs due to cardiac remodeling that results in disrupted electrical activity in the atrial tissue. This disruption causes rapid and unsynchronized atrial excitation, which leads to rhythm irregularity and fibrillation of atrial tissues [4,5]. AF can be classified as paroxysmal AF, which does not exceed seven days; persistent AF, which lasts more than a week; or long-standing persistent AF, which does not revert to the sinus rhythm for more than 12 months [6]. AF is associated with different consequences, such as peripheral vascular embolism, stroke, dementia, heart failure, and death [7]. As a result, prompt and efficient AF management is important. Treatment options include rhythm control medications, rate control medications, oral anticoagulant therapy, or atrial ablation [8].

Catheter ablation (CA) has become a reliable therapeutic option for symptomatic AF, supported by increasing evidence demonstrating its safety and effectiveness [9]. Pulmonary vein (PV) isolation (PVI) is the cornerstone of AF ablation. Although radiofrequency (RF) is still the most often utilized ablative therapy, there is an increasing interest in cryoablation therapy as a novel and potent ablative strategy that showed comparable efficacy and safety to RF with shorter procedure time and less reliance on the level of experience of the operator [10]. Cryoballon ablation is limited by the increased fluoroscopic and dye exposure throughout the procedure due to the significant anatomic diversity of PVs and the difficult PV occlusion [11-13]. A previous study reported that utilizing mapping systems in conducting cryoablation improves PVI durability and overall treatment success rate [14]. The KODEX-EPD (EPD Solutions, a Philips Company) is a recent, dielectric, three-dimensional (3D) imaging system that generates high-density voltage maps with accurate visualization of the anatomical structure of PV, which facilitates PV occlusion and improves the overall success rate of atrial ablation [14,15].

We aim in this meta-analysis to assess the efficacy, procedural characteristics, and safety of cryoablation under the guidance of the KODEX-EPD mapping system versus cryoballoon ablation in the management of patients with AF.

## Review

Methods

We followed Preferred Reporting Items for Systematic Reviews and Meta-Analyses (PRISMA) as a guideline in performing our study [16].

Search and Information

This search strategy was used till April 2024 using the following keywords: “atrial fibrillation AND ("cryoballoon ablation" OR cryoablation) AND (KODEX-EPD AND KODEX OR mapping)”. The utilized online databases were Web of Science, PubMed, Scopus, and Cochrane Library.

Selection Criteria and Eligibility Criteria

The authors screened the studies’ titles and abstracts to obtain relevant studies. After that, the selected articles underwent full-text screening based on our eligibility criteria to select the final included studies.

Study design: We included observational and controlled studies. We excluded secondary research such as meta-analyses and review articles.

Inclusion criteria: We involved studies that assessed the efficacy, procedural characteristics, and safety of cryoablation under the guidance of the KODEX-EPD mapping system versus conventional cryoballoon ablation in the management of patients with AF regarding the successful cryoballoon-based PVI, procedural duration, procedural complications, recurrence, fluoroscopic time, fluoroscopic dose, and volume of used contrast media.

Exclusion criteria: We excluded single-arm studies, studies older than 2015, and studies that did not measure our selected outcomes.

Data Extraction

We retrieved data from the eligible articles. We extracted the baseline data and the comorbidities of the included participants. Moreover, we extracted data from our selected outcomes such as successful cryoballoon-based PVI, procedural duration, procedural complications, recurrence, fluoroscopic time, fluoroscopic dose, and volume of used contrast media. We extracted data on the risk of bias.

Quality Assessment

We included both randomized controlled articles and observational studies in our meta-analysis. Thus, the risk of bias in these observational articles was assessed using the National Heart, Lung, and Blood Institute (NHLB) quality assessment tool [17]. Meanwhile, the randomized controlled study was assessed using the Cochrane quality assessment tool [18].

Statistical Methods

We extracted continuous and dichotomous outcomes. We used Review Manager (version 5.4.1; Cochrane Collaboration, London, UK) and OpenMetaAnalyst software (https://github.com/bwallace/OpenMeta-analyst-) to analyze our outcomes. Regarding the continuous data, we used mean difference (MD) and 95% confidence intervals (CIs) under the inverse variance analysis method. Risk ratio (RR) and 95% CIs under the Mantel-Haenszel analysis method were utilized in dichotomous outcomes. We used a fixed effect analysis model in homogeneous outcomes, while the random effect was used in heterogeneous outcomes. The inconsistency among the articles was measured by the I^2^ and p-value. The outcome becomes heterogeneous if P < 0.1 or I^2^ > 50%. We used the leave-one-out method in solving the heterogeneity in the volume of used contrast media outcome [19].

Results

Summary of the Included Studies

**Figure 1:**
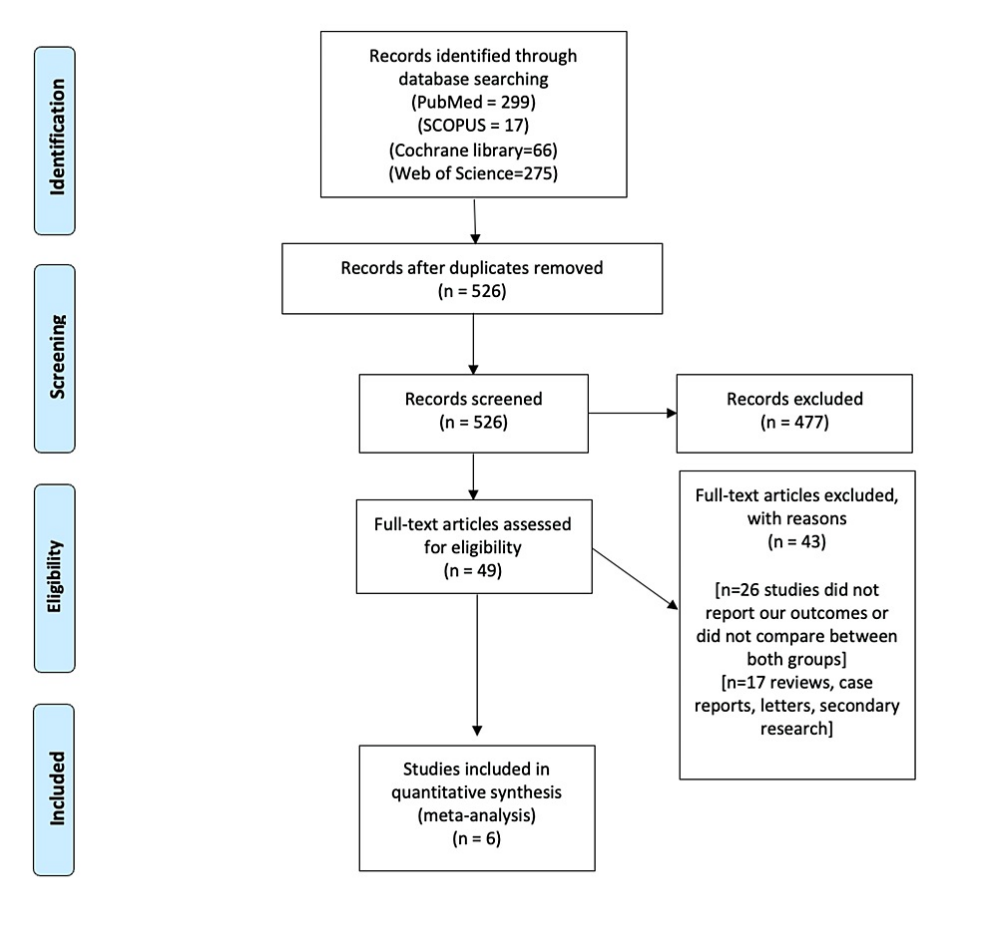
PRISMA flow chart (literature search and study selection) Following the PRISMA guidelines, our search was conducted using the academic databases PubMed, SCOPUS, Cochrane Library, and Web of Science with relevant keywords. Our search was focused on the the results of KODEX-EPD mapping compared with conventional cryoballon cryoablation throughout all specialties of medicine. We included relevant studies published over a 9-year period (2015-2024) that met the inclusion criteria of this review paper. n: number; PRISMA: Preferred Reporting Items for Systematic Reviews and Meta-Analyses

We illustrated our search results in the PRISMA diagram (Figure 1). We included six studies that were eligible to be included in our meta-analysis [20-25]. We analyzed 464 patients with AF. A total of 240 patients were treated by cryoablation guided by the KODEX-EPD mapping system, while 224 patients were treated by conventional cryoablation. Table 1 shows the demographic data and baseline characteristics of the involved participants. Table 2 shows the comorbidities of the included participants.

**Table 1:** Showing the demographic data and baseline characteristics of the involved participants Data are presented as mean ± standard deviation, number (percentage), and NA = not available.

Study ID	Sample size		Age, years		Males n (%)		BMI (kg/m^2^)		LAD, mm		CHA2DS2-VASc score		
	Mapping	Conventional	Mapping	Conventional	Mapping	Conventional	Mapping	Conventional	Mapping	Conventional	Mapping	Conventional	
Chen et al. 2024 [22]	74	74	60.50 ± 9.77	61.09 ± 10.26	51 (68.9%)	52 (70.3%)	25.67 ± 3.43	26.03 ± 3.50	39.70 ± 5.43	40.41 ± 4.62	NA	NA	
Noujaim et al. 2023 [21]	9	18	64.9	65.4	8 (88.8%)	16 (88.8%)	NA	NA	NA	NA	NA	NA	
Rottner et al. 2022 [20]	50	25	63 ± 11	66 ± 10	31 (62%)	19 (76%)	NA	NA	43 ± 7	41 ± 6	NA	NA	
Rottner et al. 2023 [23]	70	70	64 ± 13	68 ± 10	49 (70%)	43 (61.4%)	27 ± 5	27 ± 6	NA	NA	2 ±1.5	3 ± 1.5	
Schillaci et al. 2021 [24]	17	17	60 ± 8	59 ± 8	13 (76.4%)	12 (70.6%)	25.2 ± 3.5	26 ± 3.3	NA	NA	1.4 ± 0.8	1.4 ± 0.7	
Wubulikasimu et al. 2023 [25]	20	20	69.75 ± 10.66	65.10 ± 10.46	12 (60%)	15 (75%)	25.25 ± 2.69	25.65 ± 3.75	39.85 ± 6.18	40.65 ± 4.92	2.30 ± 1.46	2.20 ± 1.61	

**Table 2:** Comorbidities of the included participants Data are presented as mean ± standard deviation, number (percentage), and NA = not available.

Study ID	Paroxysmal AF		Persistent AF		Heart failure		History of stroke		Hypertension		Diabetes mellitus	
	Mapping	Conventional	Mapping	Conventional	Mapping	Conventional	Mapping	Conventional	Mapping	Conventional	Mapping	Conventional
Chen et al. 2024 [22]	44 (59.5%)	40 (54.1%)	20 (27.0%)	23 (31.1%)	1 (1.4%)	2 (2.7%)	9 (12.2%)	9 (12.2%)	30 (40.5%)	30 (40.5%)	9 (12.2%)	5 (6.8%)
Noujaim et al. 2023 [21]	NA	NA	NA	NA	NA	NA	NA	NA	NA	NA	NA	NA
Rottner et al. 2022 [20]	18 (36%)	9 (36%)	NA	NA	NA	NA	NA	NA	NA	NA	NA	NA
Rottner et al. 2023 [23]	27 (39%)	22 (31%)	43 (61%)	48 (69%)	19 (27%)	22 (31%)	5 (7%)	8 (11%)	42 (60%)	53 (76%)	4 (6%)	7 (10%)
Schillaci et al. 2021 [24]	NA	NA	NA	NA	NA	NA	NA	NA	10 (59%)	12 (70%)	NA	NA
Wubulikasimu et al. 2023 [25]	NA	NA	NA	NA	0 (0%)	1 (5%)	2 (10%)	6 (30%)	12 (60%)	13 (65%)	4 (20%)	3 (15%)

Quality Assessment Results

The mean risk of bias score of the included observational studies [20-24], according to the NHLB quality assessment tool was 10.4 out of 14, as shown in Table 3. The overall risk of bias in the only randomized controlled trial [25], according to the Cochrane Risk of Bias tool, was moderate (Table 4).

**Table 3:** Quality assessment for the included retrospective studies according to NHLB Key: 1 = Yes, 0 = No, * = Not reported, N/A = Not applicable

Q. Number	Quality Assessment Questionnaire	Chen et al. 2024 [22]	Noujaim et al. 2023 [21]	Rottner et al. 2022 [20]	Rottner et al. 2023 [23]	Schillaci et al. 2021 [24]
1.	Was the research question or objective in this paper clearly stated?	1	1	1	1	1
2.	Was the study population clearly specified and defined?	1	1	1	1	1
3.	Was the participation rate of eligible persons at least 50%?	1	1	1	1	0
4.	Were all the subjects selected or recruited from the same or similar populations (including the same time period)? Were inclusion and exclusion criteria for being in the study prespecified and applied uniformly to all participants?	1	0	1	1	1
5.	Was a sample size justification, power description, or variance and effect estimates?	0	0	0	0	0
6.	For the analyses in this paper, were the exposure (s) of interest measured prior to the outcome(s) being measured?	1	1	1	1	1
7.	Was the timeframe sufficient so that one could reasonably expect to see an association between exposure and outcome if it existed?	1	1	1	1	1
8.	For exposures that can vary in amount or level, did the study examine different levels of the exposure as related to the outcome (e.g., categories of exposure, or exposure measured as a continuous variable)?	1	1	1	1	1
9.	Were the exposure measures (independent variables) clearly defined, valid, reliable, and implemented consistently across all study participants?	1	1	1	1	1
10.	Was the exposure(s) assessed more than once over time?	1	0	0	1	0
11.	Were the outcome measures (dependent variables) clearly defined, valid, reliable, and implemented consistently across all study participants?	1	1	1	1	1
12.	Were the outcome assessors blinded to the exposure status of participants?	*	*	*	*	*
13.	Was loss to follow-up after baseline 20% or less?	1	1	1	1	1
14.	Were key potential confounding variables measured and adjusted statistically for their impact on the relationship between exposure(s) and outcome(s)?	1	0	1	0	0
	Total score (out of 14)	12/14	9/14	11/14	11/14	9/14

**Table 4:** Quality assessment for the included randomized controlled study according to the Cochrane tool

Study	Randomization	Allocation concealment	Blinding of participants and personnel	Blinding of outcome assessment	Attrition bias	Selective reporting	Other bias
Wubulikasimu et al. 2023 [25]	Low	High	High	High	Low	Low	Low

Analysis of Outcomes

**Figure 2:**
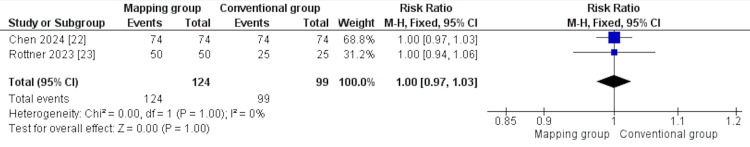
Successful Cryoballoon-based PVI [22, 23]

Successful cryoballoon-based PVI: Two studies [22,23] reported this outcome. Both groups showed successful PVI in all included patients without any difference between both cohorts (RR = 1.00 (0.97, 1.03); P = 1.00). Data were homogeneous (P = 1.00); I² = 0% (Figure 2).

**Figure 3:**
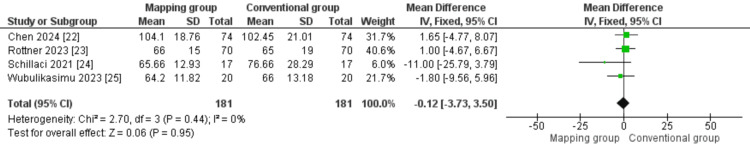
Procedural duration Sources: [22-25]

Procedural duration (min): A total of 362 patients from four studies [22-25] were analyzed regarding the total procedural time. Our analysis showed similar procedure times in both groups (MD = -0.12 (-3.73, 3.50); P = 0.95). The overall analysis was homogeneous (P = 0.44; I² = 0%) (Figure 3).

**Figure 4:**
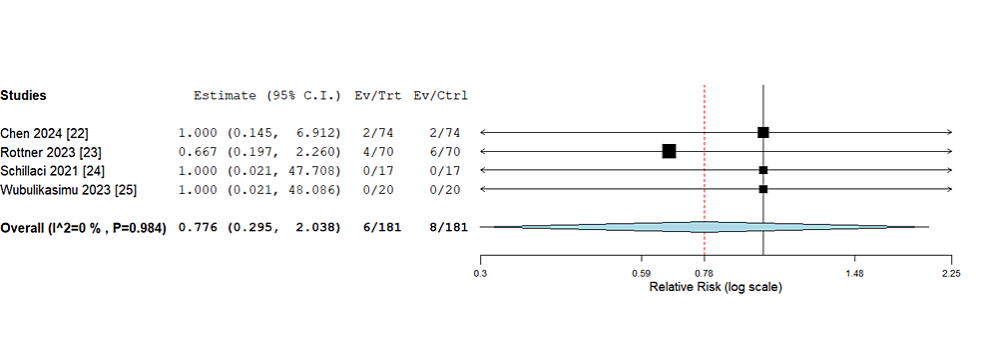
Procedural complications Sources: [22-25]

Procedural complications: Four studies [22-25] evaluated the incidence of each procedure-related complication. The incidence of procedural complication was comparable in both groups, with an overall RR = 0.776 (0.295, 2.038) and P = 0.607. Pooled analysis was homogeneous (P = 0.984; I² = 0%; Figure 4).

**Figure 5:**
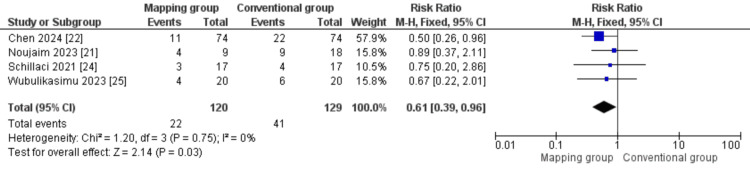
Recurrence Sources: [21,22,24,25]

Recurrence: We analyzed 249 patients from four studies [21,22,24,25] investigating the risk of recurrence after each procedure. Our analysis showed a significantly lower incidence of recurrence in patients who underwent cryoablation guided by the KODEX mapping system (RR = 0.61 (0.39, 0.96), P = 0.03). The overall analysis was homogeneous (P = 0.75; I² = 0%; Figure 5).

**Figure 6:**
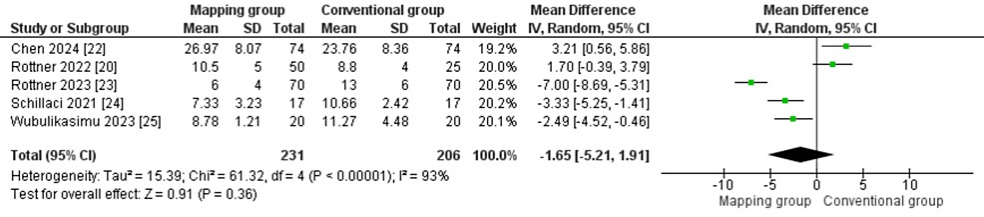
Fluoroscopic time Sources: [20,22-25]

Fluoroscopic time (min): The majority of included studies [20,22-25] assessed the fluoroscopic time. The combined MD showed similar fluoroscopic times in both groups (MD = -1.65 (-5.21, 1.91), P = 0.36). We observed the heterogeneity among data in this outcome (P < 0.001; I² = 93%; Figure 6).

**Figure 7:**
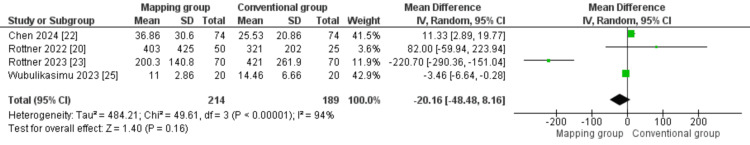
Fluoroscopic dose Sources: [20,22,23,25]

Fluoroscopic dose (cGy.cm2): Four studies [20,22,23,25] reported the utilized fluoroscopic doses in each group. We found no significant variation among both groups (MD = -20.16 (-48.48, 8.16), P = 0.16). Data of this outcome were heterogeneous (P < 0.001; I² = 94%; Figure 7).

**Figure 8:**
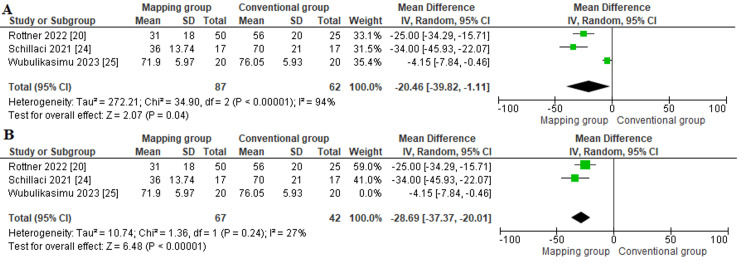
Contrast volume Sources: [20,24,25]

Volume of used contrast media (mL): The volume of utilized contrast media was reported in three studies [20,24,25]. We found that patients allocated to the mapping group required a significantly lower contrast volume than those who were allocated to the conventional cryoablation (MD = -20.46 (-39.82, -1.11), P = 0.04). The overall analysis was heterogeneous (P < 0.001; I² = 94%; Figure 8a). However, we could solve the heterogeneity by excluding Wubulikasimu et al. (P = 0.24; I² = 27%) [25]. The combined mean difference after solving the heterogeneity also showed a significantly lower contrast volume in the mapping group (MD = -28.69 (-37.37, -20.01), P < 0.001) (Figure 8b).

Discussion

It is well-known that there are various excitable points that are located in the PVs and the junction between the PVs and the left atrium. These excitable tissues have a major role in the development of AF. Thus, the cornerstone of the management of AF not responding to antiarrhythmic medication is disrupting the electrical signals between the excitable tissues in the PVs and the atrial tissues by atrial ablation techniques [26]. The ablative methods aim to completely isolate and disconnect these PVs from the rest of the atrial tissue [27]. Despite the considerable technological advancements in ablation therapies such as involving catheters with irrigating tips and catheters with a measured contact force, there are various complications in these procedures. Overheating of the excitable tissues by radiofrequency may injure cardiac and extracardiac structures, yielding major consequences [28]. In this context, cryoballoon ablation using connective cooling has gathered evidence and achieved popularity to be regarded as an effective substitute for radiofrequency [10]. Cryoablation guided by 3D mapping systems is associated with better procedural guidance and better detection of low-voltage areas, which improve the overall success rate and decrease the incidence of AF recurrence [15,29].

This is the first meta-analysis that compares the efficacy, procedural characteristics, and safety of cryoablation under the guidance of the KODEX-EPD mapping system versus conventional cryoballoon ablation in the management of patients with AF. We found that cryoablation guided by the KODEX-EPD system was associated with a lower recurrence rate after the procedure. Furthermore, it allowed a significant reduction in the volume of contrast medium used during the procedure when compared to the conventional cryoablation. However, our findings showed no significant difference between both procedures in terms of successful cryoballoon-based PVI, procedural duration, procedural complications, fluoroscopic time, and fluoroscopic dose. The KODEX-EPD mapping system provides high-resolution 3D cardiac imaging and creates real-time voltage and activation maps, which allows locating the PVs and the associated accessory structures more precisely enhancing the overall success rates of the procedure [25]. Isolating the superior and inferior PVs at the same time is not accessible for cryoablation due to the size mismatch between the PV ostium and the cryoballoon catheter, in addition to its spherical nature. This results in ignoring a thick segment existing between the ipsilateral PVs called carina [30]. Previous studies reported that additional carina ablation may be needed in a considerable percentage of patients to make sure that all PV potentials were eliminated [31]. Mapping-guided cryoablation can provide high-density voltage mapping that detects any residual excitable tissues in the carina area, yielding an overall decrease in the risk of post-procedural recurrence [29,31].

A recent study in 2024 by Chen et al. [22] evaluated the one-year outcomes of patients who underwent cryoablation guided by the KODEX-EPD system versus patients who were treated by conventional cryoballoon ablation procedure. They reported that both mapping and conventional procedures were associated with comparable successful PVI, procedure duration, and procedure complications, which is consistent with our findings. However, they reported higher fluoroscopic exposure and longer fluoroscopic time in the mapping cohort. They attributed this prolonged fluoroscopic duration to the greater number of cryoapplications in the mapping group to guarantee complete PVI [32]. Schillaci et al. investigated patients with refractory AF not responding to more than one antiarrhythmic drug [24]. Patients were treated by either KODEX-EPD-guided cryoablation or conventional non-mapping cryoablation. This study demonstrated similar success rates, procedure time, procedure complications, and arrhythmia recurrence in both procedures. The mapping group allowed a reduction of the dye use and fluoroscopic time. These findings were in line with a more recent retrospective study [21] which reported a similar recurrence rate in both techniques. This study also showed a significant favoring of the KODEX-EPD system regarding the successful PVI and the ablation scar formation in patients with AF. In 2022, a previous study of 75 patients with AF undergoing cryoballoon-based PVI compared cryoballon using the KODEX-EPD tool with conventional cryoablation. They found comparable fluoroscopy times P=0.23 and fluoroscopic doses P=0.44 in both groups. They also found that patients allocated to the mapping group required a significantly reduced amount of contrast dye. These findings are supported by our results [20]. Wubulikasimu et al. found that the KODEX-EPD mapping system could decrease the contrast agent and fluoroscopic exposure significantly. As a result, it can prevent renal or cardiac deterioration in patients with cardiovascular and renal diseases who are contraindicated to perform the conventional cryoablation [25].

Previous studies reported various advantages of the KODEX system compared to other mapping systems such as the panoramic view (PANO View), which helps the planning of ablation strategies by illustrating the endocardial surface. PANO view also aids in reducing X-ray exposure. Additionally, by using integrated tissue-pressure technology, the KODEX system allows real-time contact force monitoring without raising costs [33]. A previous study compared high-density KODEX‐EPD mapping versus 3D computed tomography (CT) imaging in mapping the left atrium. This study concluded that the KODEX-EPD system provides an accurate and fast mapping technique with high reliability when utilized in cases of atrial ablation [34].

Our study is the first and most recent meta-analysis evaluating the success rate, safety profile, and procedural characteristics of cryoballoon ablation guided by the KODEX-EPD mapping system versus conventional cryoballon ablation in the management of patients with AF. The main limitations of our study are the relatively small sample size and the heterogeneity of some outcomes. Additionally, we did not consider the operator experience in our study, which may affect the overall success rates and complications of both procedures.

## Conclusions

The use of the novel KODEX-EPD mapping system in a cryoablation procedure was associated with a significant reduction of the volume of contrast medium use and the recurrence rate compared with the conventional cryoablation while preserving similar efficacy, safety profile, and procedure time. However, further randomized studies with larger sample sizes are needed to confirm our findings.
